# The Influence of the COVID 19 Pandemic on Food Insecurity Among Cancer Survivors Across New York State

**DOI:** 10.1007/s10900-024-01358-1

**Published:** 2024-04-29

**Authors:** Marlene Camacho-Rivera, Katherine Haile, Eshani Pareek, Debra D’Angelo, Francesca Gany, Francesca Maglione, Kellie Jack, Alexina Cather, Erica Phillips

**Affiliations:** 1 Department of Community Health Sciences, School of Public Health, SUNY Downstate Health Sciences University, Brooklyn, NY USA; 2 Bureau of Chronic Disease Evaluation and Research, New York State Department of Health, Albany, NY USA; 3 Weill Cornell Medicine Division of Biostatistics, Population Health Sciences, New York, NY USA; 4 Immigrant Health & Cancer Disparities Service, Department of Psychiatry & Behavioral Sciences, Department of Medicine, Memorial Sloan-Kettering Cancer Center, New York, NY USA; 5 Department of Food and Nutrition, NewYork-Presbyterian/Weill-Cornell Medical Center, New York, NY USA; 6 Sandra and Edward Meyer Cancer Center, Office of Community Outreach and Engagement, Weill Cornell Medicine, New York, NY USA; 7 Wellness in the Schools, New York, NY USA; 8 Center for Food as Medicine, New York, NY USA; 9 Division of General Internal Medicine, Department of Medicine, Weill Cornell Medicine, 338 East 66th Street, Box #46, New York, NY 10021 USA

**Keywords:** Food insecurity, Cancer, Social determinants, Disparities, COVID-19

## Abstract

People surviving cancer represent a particularly vulnerable population who are at a higher risk for food insecurity (FI) due to the adverse short- and long-term effects of cancer treatment. This analysis examines the influence of the COVID-19 pandemic on the prevalence of FI among cancer survivors across New York State (NYS). Data from the 2019 and 2021 NYS Behavioral Risk Factor Surveillance System (BRFSS) were used to estimate the prevalence of FI. Multivariable logistic regression was used to explore socioeconomic determinants of FI. Among cancer survivors, FI varied geographically with a higher prevalence in New York City compared to the rest of the state (ROS) prior to (25.3% vs. 13.8%; *p* = .0025) and during the pandemic (27.35% vs. 18.52%; *p* = 0.0206). In the adjusted logistic regression model, pre-pandemic FI was associated with non-White race (OR 2.30 [CI 1.16–4.56]), household income <$15,000 (OR 22.67 [CI 6.39–80.43]) or $15,000 to less than <$25,000 (OR 22.99 [CI 6.85–77.12]), and more co-morbidities (OR 1.39 [CI 1.09–1.77]). During the pandemic, the association of FI with non-White race (OR 1.76 [CI 0.98–3.16]) was attenuated but remained significant for low household income and more co-morbidities. FI was newly associated with being out of work for less than one year (OR 6.36 [CI 1.80–22.54] and having one (OR 4.42 [CI 1.77–11.07]) or two or more children in the household (OR 4.54 [CI 1.78–11.63]). Our findings highlight geographic inequities and key determinants of FI among cancer survivors that are amendable to correction by public health and social policies, for which several were momentarily implemented during the pandemic.

## Background

As defined by the United States Department of Agriculture, food insecurity is a lack of consistent access to enough food for every person in a household to live an active, healthy life. This can be a temporary situation for a family or can last a long time. In 2019, the year that preceded the Coronavirus Disease (COVID-19) pandemic, an estimated 10.5% of US households (down from 11.1% in 2018) were food insecure at least sometime during the prior year [1]. Historically, rates of food insecurity have been higher than the national average for the following groups: households with incomes near or below the federal poverty level ($14,580 for a household size of 1) [2]; all households with children and particularly households with children headed by single women or single men; women and men living alone; Black- and Hispanic-headed households; and households in principal cities and nonmetropolitan areas. The prevalence of food insecurity also varies by state, ranging from 6.6% in New Hampshire to 15.7% in Mississippi in 2017–2019. Food insecurity rates across New York State (NYS) during this same 3-year period were similar to the national average (10.8%) [3].

The COVID-19 pandemic caused widespread economic hardships and disrupted food systems across the country, disproportionately impacting the most vulnerable populations, especially in states hardest hit by the pandemic, such as New York. People surviving cancer represent a particularly vulnerable population secondary to their higher oncological risk (recurrent or secondary cancer). Food insecurity may be more prevalent in cancer survivors due to the high cost of treatment, lost wages, unemployment, and increased burden placed on caregivers within the same household [4]. Additionally, among those with a cancer diagnosis, food insecurity has been associated with decreased adherence to cancer treatment, foregone medical care, increased mental health issues such as depression, and adverse physical health outcomes. To our knowledge, the impact of the COVID-19 pandemic on food access has not been assessed among cancer survivors across NYS. Hence, this analysis is the first to (1) describe the prevalence of food insecurity amongst cancer survivors before (2019) and during the COVID pandemic (2021) across the state, as well as any geographic variability; (2) examine demographic and socioeconomic determinants associated with food insecurity among cancer survivors during both periods.

## Methods

We used publicly available data from the NYS Behavioral Risk Factor Surveillance System (BRFSS) for 2019 and 2021. BRFSS is sponsored by the Centers for Disease Control and Prevention and is an annual telephone survey administered by individual states to collect state-specific data on health-related risk behaviors, chronic health conditions, and the use of preventive services among the noninstitutionalized civilian population aged 18 years and older [5]. Interviews are conducted throughout the year in both English and Spanish and reach households with landline telephones or cell phones. The BRFSS allows for a sub-division of analysis to compare respondents residing in NYC (five boroughs combined) to residents in the rest of the state (ROS). The data within the BRFSS data set are neither identifiable nor private and thus do not meet the federal definition of “human subject” as defined in 45 CFR 46.102. Therefore, this study did not need to be reviewed and approved by the Institutional Review Board.

The 2021 annual NYS BRFSS was an expanded BRFSS [6] designed to support regional and county-level analysis, resulting in a larger sample size. The BRFSS uses a core set of questions and allows states to include additional questions to serve their specific needs [7]. Food security was a NYS-Added Module in the 2019 and 2021 BRFSS surveys. The module included the following question: *How often in the past 12 months would you say you were worried or stressed about having enough money to buy nutritious meals? Would you say—always, usually, sometimes, rarely, never?* Food insecure individuals were defined as respondents who indicated they were always, usually, or sometimes worried or stressed about having enough money to buy nutritious meals in the past 12 months. Cancer survivors were defined as respondents who indicated they were ever told by a doctor, nurse, or other health professionals that they had any type of cancer other than skin cancer. Respondents only reporting a skin cancer diagnosis were excluded as the survey does not distinguish between melanoma and nonmelanoma skin cancers, and nonmelanoma skin cancer typically does not require treatment beyond surgery [8].

## Statistical Analysis

All statistical analyses were performed in SAS version 9.4 software (Cary, NC, USA). Considering the complex survey design of BRFSS, all statistical analyses incorporated stratification and weighting following the methodology established by the Centers for Disease Control and Prevention [9, 10]. We computed unweighted and survey-weighted descriptive statistics to summarize the demographic and clinical characteristics of respondents in 2019 and 2021 based on cancer survivorship status, then used SAS proc surveyfreq to perform survey-weighted chi-squared tests comparing characteristics among four cohorts: 2019 cancer survivors (A), 2019 respondents without cancer (B), 2021 cancer survivors (C), and 2021 respondents without cancer (D). We performed an additional survey-weighted chi-squared analysis in the subset of cancer survivors to compare the geographic distribution of food insecurity in the following four cohorts: 2019 NYC (A), 2019 ROS (B), 2021 NYC (C), and 2021 ROS (D). We then performed univariable logistic regression models using SAS proc surveylogistic to identify factors associated with food insecurity in 2019 and 2021. We chose the most scientifically and evidence-based relevant variables with a significance of *p* < 0.10 in the univariable models to include in multivariable models predicting food insecurity among cancer survivors in 2019 and 2021.

### Covariates

The following covariates were included in the multivariable analyses: age (18–64, 65+), sex at birth (male, female), self-identified race (White, Others), education (less than high school, high school or higher), income (<$15,000, $15,000 to less than $25,000, $25,000 to less than $50,000, or $50,000 or more), employment status (employed for wages, self-employed, out of work less than 1 year, retired, or unemployed), marital status (married/member of an unmarried couple, widowed/divorced/separated, or never married), number of children (one child, two or more children, no children), health insurance (Medicaid, Medicare, private plan, other insurance, or no coverage) and total number of chronic health conditions (0–7). The chronic conditions included have been previously associated with an increased burden of food insecurity and include diabetes, myocardial infarction, angina/coronary heart disease, asthma, chronic obstructive pulmonary disease, depressive disorder, or kidney disease [11–15].

## Results

Respondents who did not answer the question regarding a cancer diagnosis were excluded from the analysis, resulting in 14,182 respondents in 2019 and 38,927 respondents in 2021. Tables 1 and 2 depict the characteristics of the respondents to the BRFSS survey in 2019 and 2021, stratified by those who met the criteria of being a cancer survivor versus those who never had a cancer diagnosis. In both years, cancer survivors were more often older (57.4% vs. 18.6%; 60.1% vs. 19.9% *p* < 0.0001), female (61.6% vs. 51.3%; 60.8% vs. 51.4% *p* < 0.0001), self-identified as white (70.9% vs. 54.4%; 75.9% vs. 55.0% *p* < 0.0001), disabled (41.1% vs. 24.8%; 41.8% vs. 25.4% *p* < 0.0001), retired (44.5% vs. 16.31%; 49.5% vs. 17.41% *p* < 0.0001), divorced/separated or widowed (31.0% vs. 17.2%; 31.4% and 17.1%; *p* < 0.0001), resided with no children in the household (86.4% vs. 63.9%; and 86.1% vs. 65.8% *p* < 0.0001), and had Medicare as their primary health insurance (43.5% vs. 16.2%; 49.7% vs. 19.1% *p* < 0.0001). Regarding cancer risk factors, cancer survivors were more often former smokers (38.4% vs. 22.4%; 37.6% vs. 19.9% *p* < 0.0001) and less physically active (32.3% vs. 26.8%; 32.1% vs. 25.3% *p* < 0.0001). They also self-reported a higher proportion of other chronic health conditions, including diabetes (19.5% vs. 11%; 20.5% vs. 11.9%; *p* < 0.0001), myocardial infarction (9.6% vs. 3.5%; 8.0% vs. 3.2%; *p* < 0.0001), angina/CHD (9.1% vs. 3.5%; 9.1% vs. 3.6% *p* < 0.0001), chronic obstructive lung disease (13.94% vs. 5.11%; 13.69% vs. 4.88% *p* < 0.0001), depression (18.2% vs. 15.1% *p* = 0.0308; 21% vs. 16.7%; *p* = 0.0010) and kidney disease (7.5% vs. 2.1%; 8.2% vs. 2.4%; *p* < 0.0001).

**Table 1:** Characteristics of New York State (NYS) BRFSS survey respondents by cancer survivorship status in 2019 (*N* = 14,182)

	Cancer survivors (*n* = 1422)			Respondents without a cancer diagnosis (*n* = 12,760)			*p*-Values
	Unweighted Freq	Weighted Freq	Weighted percent (95% CI)	Unweighted Freq	Weighted Freq	Weighted percent (95% CI)	
Age (years)							
18–64	454	440,820	42.58 (38.68, 46.48)	8370	11,377,145	81.37 (80.54, 82.2)	<0.0001
65 or older	938	594,395	57.42 (53.52, 61.32)	4028	2,604,987	18.63 (17.8, 19.46)	
Sex at birth							
Female	851	652,334	61.63 (57.89, 65.37)	6785	7,364,693	51.32 (50.05, 52.6)	<0.0001
Male	571	406,136	38.37 (34.63, 42.11)	5975	6,984,864	48.68 (47.4, 49.95)	
Race/ethnicity							
White	1145	726,433	70.95 (67.01, 74.88)	8618	7,565,249	54.41 (53.17, 55.64)	<0.0001
Black	96	109,628	10.71 (7.9, 13.52)	1191	1,987,624	14.29 (13.33, 15.25)	
Hispanic	81	133,580	13.05 (9.97, 16.12)	1534	2,694,311	19.38 (18.32, 20.43)	
Multiracial, non-Hispanic	19	13,859	1.35 (0.53, 2.18)	180	100,593	0.72 (0.58, 0.87)	
Other race, non-Hispanic	40	40,438	3.95 (2.04, 5.85)	846	1,557,338	11.2 (10.17, 12.23)	
Education							
Less than high school grade	103	136,459	12.97 (9.95, 15.99)	974	1,946,119	13.69 (12.66, 14.72)	0.6625
High school grad or higher	1310	916,002	87.03 (84.01, 90.05)	11,675	12,270,235	86.31 (85.28, 87.34)	
Disability status^a^	560	408,138	41.01 (37.15, 44.86)	3356	3,287,636	24.89 (23.77, 26.02)	<0.0001
Income							
<$15,000	98	83,734	10.65 (7.7, 13.6)	902	989,630	9.13 (8.29, 9.98)	0.2387
$15,000 to less than $25,000	188	112,140	14.26 (11.21, 17.31)	1687	1,832,745	16.92 (15.83, 18)	
$25,000 to less than $50,000	273	158,813	20.2 (17.03, 23.37)	2150	2,179,399	20.12 (18.95, 21.28)	
$50,000 or more	536	431,500	54.89 (50.58, 59.19)	5171	5,831,982	53.83 (52.39, 55.27)	
Employment status							
Employed for wages	304	276,314	26.56 (23.12, 30.01)	5329	6,668,291	47.88 (46.60, 49.17)	<0.0001
Self-employed	98	77,503	7.45 (5.39, 9.51)	1234	1,505,311	10.81 (10.00, 11.61)	
Out of work for less than 1 year	14	11,461	1.10 (0.18, 2.03)	309	467,759	3.36 (2.85, 3.87)	
Retired	741	462,894	44.50 (40.70, 48.29)	3414	2,271,382	16.31 (15.50, 17.12)	
Unemployed	241	212,041	20.38 (17.17, 23.59)	2171	3,013,134	21.64 (20.50, 22.78)	
Marital status							
Never married	175	131,850	12.57 (10.09, 15.05)	2814	4,249,559	30.04 (28.80, 31.29)	<0.0001
Married/living as married	646	591,642	56.40 (52.66, 60.14)	6348	7,467,373	52.79 (51.51, 54.08)	
Divorced/widowed/separated	590	325,545	31.03 (27.64, 34.42)	3423	2,427,174	17.16 (16.31, 18.01)	
No. of Children in the household							
0	1256	899,400	86.43 (83.76, 89.09)	9127	8,861,644	63.93 (62.64, 65.22)	<0.0001
1	78	84,210	8.09 (5.85, 10.33)	1460	2,100,818	15.16 (14.16, 16.15)	
2 or more	65	57,048	5.48 (3.87, 7.10)	1843	2,898,671	20.91 (19.79, 22.03)	
Primary health insurance							
Private plan	465	385,702	37.97 (34.18, 41.77)	5821	6,701,793	50.64 (49.32, 51.95)	<0.0001
Medicare	697	441,531	43.47 (39.71, 47.23)	3075	2,130,804	16.1 (15.26, 16.94)	
Medicaid	114	109,746	10.8 (8.2, 13.41)	1406	1,894,920	14.32 (13.36, 15.28)	
Other insurance	78	50,035	4.93 (3.34, 6.51)	651	713,251	5.39 (4.79, 5.99)	
No coverage	38	28,699	2.83 (1.71, 3.94)	1095	1,794,025	13.56 (12.55, 14.56)	
Smoking status							
Current smoker—every day	126	96,239	9.68 (7.38, 11.97)	1078	1,075,825	8.18 (7.49, 8.88)	<0.0001
Current smoker—some days	33	18,380	1.85 (1.04, 2.66)	471	594,880	4.52 (3.93, 5.12)	
Former smoker	539	382,638	38.48 (34.67, 42.28)	3255	2,945,654	22.4 (21.36, 23.44)	
Never smoked	645	497,202	50 (46.07, 53.92)	7040	8,534,651	64.9 (63.67, 66.13)	
Heavy drinking^b^	102	101,991	10.76 (7.75, 13.76)	1736	2,168,077	17.21 (16.19, 18.23)	0.0008
No exercise^c^	420	314,581	32.33 (28.65, 36.01)	3109	3,431,670	26.77 (25.57, 27.96)	0.0033
Presence of comorbidities							
Diabetes	293	205,974	19.49 (16.59, 22.39)	1668	1,571,880	10.98 (10.20, 11.76)	<0.0001
Myocardial Infarction (MI)	144	100,515	9.58 (7.34, 11.82)	609	492,647	3.46 (3.03, 3.9)	<0.0001
Angina/coronary heart disease (CHD)	146	94,116	9.06 (6.7, 11.43)	599	493,835	3.47 (2.99, 3.95)	<0.0001
Asthma	219	180,761	17.19 (14.10,20.28)	1856	2,083,924	14.57 (13.68,15.45)	0.0900
Chronic obstructive lung disease	225	146,901	13.94 (11.41, 16.47)	976	729,630	5.11 (4.62, 5.6)	<0.0001
Depressive disorder	276	192,162	18.18 (15.32, 21.03)	2104	2,146,142	15.06 (14.15, 15.97)	0.0308
Kidney disease	111	77,885	7.46 (5.61, 9.31)	373	301,412	2.11 (1.8, 2.41)	<0.0001
Food Insecure	171	149,172	17.78 (14.31, 21.25)	1683	2,033,389	19.5 (18.31, 20.69)	0.3716

^a^Respondents who reported at least one type of disability (cognitive, self-care, independent living, vision, mobility or hearing)

^b^Heavy drinking is defined as consuming 8 or more drinks per week for women and 15 or more drinks per week for men

^c^Leisure time physical activity is defined as any physical activities or exercises engaged in not related to one’s regular job

**Table 2:** Characteristics of New York State (NYS) BRFSS survey respondents by cancer survivorship status in 2021 (*N* = 38,297)

	Cancer survivors (*n* = 3897)			Respondents without a cancer diagnosis (*n* = 35,030)			*p*-Values
	Unweighted Freq	Weighted Freq	Weighted percent (95% CI)	Unweighted Freq	Weighted Freq	Weighted percent (95% CI)	
Age (years)							
18–64	1198	390,846	39.87 (36.81, 42.93)	22,498	11,644,783	80.1 (79.43, 80.77)	<0.0001
65 or older	2606	589,469	60.13 (57.07, 63.19)	11,382	2,892,912	19.9 (19.23, 20.57)	
Sex at birth							
Female	2343	613,404	60.84 (57.96, 63.73)	18,684	7,705,418	51.46 (50.53, 52.39)	<0.0001
Male	1554	394,765	39.16 (36.27, 42.04)	16,346	7,267,831	48.54 (47.61, 49.47)	
Race/ethnicity							
White	3302	747,907	75.96 (73.08, 78.84)	25,096	7,988,311	55.07 (54.18, 55.96)	<0.0001
Black	169	98,347	9.99 (8.02, 11.96)	2720	2,068,126	14.26 (13.59, 14.92)	
Hispanic	139	90,855	9.23 (7.17, 11.28)	3441	2,717,702	18.74 (18.02, 19.45)	
Multiracial, non-Hispanic	83	6894	0.7 (0.42, 0.98)	666	119,226	0.82 (0.69, 0.95)	
Other race, non-Hispanic	107	40,605	4.12 (2.53, 5.72)	2112	1,612,294	11.11 (10.39, 11.84)	
Education							
Less than high school grad	207	87,181	8.7 (6.87, 10.53)	2528	1,939,708	13.07 (12.36, 13.77)	0.0002
High school grad or higher	3674	914,904	91.3 (89.47, 93.13)	32,265	12,906,098	86.93 (86.23, 87.64)	
Disability status^a^	1,626	394,585	41.83 (38.74, 44.93)	9450	3,475,575	25.48 (24.63, 26.32)	<0.0001
Income							
<$15,000	200	43,057	6.08 (4.46, 7.7)	1759	754,172	7 (6.45, 7.55)	0.1345
$15,000 to less than $25,000	378	68,557	9.68 (7.89, 11.47)	3128	1,231,727	11.43 (10.74, 12.12)	
$25,000 to less than $50,000	925	206,109	29.1 (25.83, 32.37)	7255	2,693,043	24.99 (24.07, 25.92)	
$50,000 or more	1409	390,529	55.14 (51.63, 58.65)	14,102	6,095,862	56.58 (55.52, 57.63)	
Employment status							
Employed for wages	736	234,038	23.69 (20.96, 26.42)	14,314	6,904,709	47.74 (46.79, 48.68)	<0.0001
Self-employed	210	71,161	7.20 (5.60, 8.80)	2973	1,363,163	9.42 (8.88, 9.97)	
Out of work for less than 1 year	53	28,089	2.84 (1.29, 4.39)	957	515,909	3.57 (3.21, 3.93)	
Retired	2283	489,018	49.50 (46.48, 52.51)	10,055	2,518,357	17.41 (16.78, 18.05)	
Unemployed	559	165,703	16.77 (14.55, 18.99)	5809	3,162,003	21.86 (21.03, 22.69)	
Marital status							
Never married	396	115,326	11.61 (9.72, 13.51)	7313	4,386,832	29.72 (28.83, 30.61)	<0.0001
Married/living as married	1934	566,022	56.98 (54.05, 59.92)	18,132	7,849,858	53.18 (52.25, 54.12)	
Divorced/widowed/separated	1531	311,939	31.40 (28.74, 34.07)	9099	2,523,431	17.10 (16.46, 17.74)	
Number of children in the household							
0	3479	845,853	86.11 (83.69, 88.54)	25,231	9,473,339	65.76 (64.84, 66.68)	<0.0001
1	187	76,974	7.84 (5.79, 9.89)	3746	2,119,585	14.71 (14.01, 15.42)	
2 or more	162	59,422	6.05 (4.55, 7.55)	4919	2,813,349	19.53 (18.76, 20.30)	
Primary health insurance							
Private plan	1079	319,086	33.15 (30.14, 36.15)	14,810	6,847,125	49.34 (48.38, 50.3)	<0.0001
Medicare	2030	478,234	49.68 (46.61, 52.75)	9519	2,653,110	19.12 (18.42, 19.82)	
Medicaid	286	81,840	8.5 (6.84, 10.16)	3641	2,020,901	14.56 (13.85, 15.28)	
Other insurance	284	72,083	7.49 (5.85, 9.13)	3509	1,471,992	10.61 (10.01, 11.2)	
No coverage	43	11,391	1.18 (0.63, 1.74)	1433	883,160	6.36 (5.89, 6.84)	
Smoking status							
Current smoker—every day	325	66,057	6.55 (5.31, 7.80)	3400	1,081,328	7.22 (6.76, 7.68)	<0.0001
Current smoker—some days	120	31,401	3.11 (2.17, 4.06)	1295	569,617	3.80 (3.46, 4.15)	
Former smoker	1521	378,622	37.56 (34.68, 40.43)	8959	2,977,084	19.88 (19.18, 20.58)	
Never smoked	1715	465,715	46.19 (43.20, 49.19)	18,543	8,944,165	59.73 (58.83, 60.64)	
Heavy drinking^b^	232	60,355	6.66 (5.1, 8.22)	4272	2,013,629	15.47 (14.73, 16.2)	<0.0001
No exercise^c^	1271	323,586	32.12 (29.35, 34.9)	8717	3,782,815	25.33 (24.51, 26.15)	<0.0001
Presence of comorbidities							
Diabetes	883	206,654	20.50 (18.16, 22.84)	4884	1,775,625	11.89 (11.28, 12.50)	<0.0001
Myocardial Infarction (MI)	398	79,468	7.99 (6.62, 9.35)	1689	476,727	3.21 (2.91, 3.52)	<0.0001
Angina/coronary heart disease (CHD)	418	90,688	9.11 (7.48, 10.75)	1705	527,642	3.55 (3.23, 3.87)	<0.0001
Asthma	621	145,560	14.48 (12.49,16.47)	5090	2,112,386	14.16 (13.50,14.82)	0.7640
Chronic obstructive lung disease	661	137,561	13.69 (11.72, 15.66)	2678	726,779	4.88 (4.48, 5.28)	<0.0001
Depressive disorder	865	206,071	20.54 (18.21, 22.87)	6816	2,485,946	16.74 (16.08, 17.41)	0.0010
Kidney disease	348	81,696	8.15 (6.36, 9.94)	1171	360,975	2.42 (2.15, 2.69)	<0.0001
Food Insecure	362	177,161	20.36 (17.15, 23.56)	3907	3,143,774	25.12 (24.04, 26.19)	0.0061

^a^Respondents who reported at least one type of disability (cognitive, self-care, independent living, vision, mobility or hearing)

^b^Heavy drinking is defined as consuming 8 or more drinks per week for women and 15 or more drinks per week for men

^c^Leisure time physical activity is defined as any physical activities or exercises engaged in not related to one’s regular job

Prior to the COVID pandemic, the proportion of cancer survivors across the state experiencing food insecurity in comparison to respondents with no prior cancer diagnosis did not significantly differ (17.78% vs. 19.5%; *p* = 0.3716). However, food insecurity among cancer survivors varied geographically, with a higher prevalence in the five boroughs of NYC compared to the ROS (25.3% vs. 13.8%; *p* = 0025), as shown in Table 3. While food insecurity rates increased across the state because of the pandemic, the geographic difference among cancer survivors persisted (27.35% vs. 18.52%; *p* = 0.0206).

**Table 3:** Food insecurity—NYC vs. Rest of State (ROS), 2019 and 2021

	Cancer survivors 2019					Cancer survivors 2021				
	Unweighted Freq	Weighted Freq	Weighted percent	95% CI	*p*-Value	Unweighted Freq	Weighted Freq	Weighted percent	95% CI	*p*-Value
New York City	53	73,520	25.26	17.52–33.01	0.0025	61	49,581	27.35	20.29–34.42	0.0206
Rest of the state	118	75,653	13.81	10.57–17.04		301	127,580	18.52	15.05–21.98	

The multivariable survey-weighted logistic regression models predicting food insecurity among cancer survivors in both years are shown in Table 4. Pre-pandemic cancer survivors had higher odds of being food insecure if they self-identified as non-White race (Odds Ratio 2.30 [95% Confidence Interval, 1.16, 4.56], had a household income <$15,000 (22.67 [6.39, 80.43]) or $15,000 to less than <$25,000 (22.99 [6.85, 77.12]), and had more cumulative chronic health conditions (1.39 [1.09, 1.77]). During the pandemic, the odds of being food insecure among cancer survivors were attenuated for non-White race (1.76 [0.98–3.16]) but remained significant for those with a household income <$15,000 (15.25 [5.3–43.91]) or $15,000 to less than <$25,000 (OR 11.81 [4.80–29.06]) as well as those with more co-morbidities (1.56 [1.28, 1.90]). Cancer survivors out of work for less than one year (6.36 [1.80, 22.54) and those with children in the household (one child: 4.42 [1.77, 11.07]; two or more children; 4.54 [1.78, 11.63]) also had a higher odd of being food insecure. The odds of food insecurity were lower among cancer survivors with private health insurance (0.20 [0.06, 0.64]) compared to no health insurance.

**Table 4:** Determinants of food insecurity among cancer survivors across NYS State in 2019 and 2021

	2019			2021		
	Cancer survivors (*N* = 897)			Cancer survivors (*N* = 1631)		
	OR (95% CI)	*p*-Values		OR (95% CI)	*p*-Values	
		Level	Overall		Level	Overall
Age (*18–64 vs. 65 years or older*)	1.49 (0.55, 4.03)	–	0.430	0.88 (0.42, 1.86)	–	0.735
Sex (*Female vs. male*)	1.02 (0.50, 2.07)	–	0.964	1.23 (0.73, 2.07)	–	0.440
Race (*Others vs. White*)	2.30 (1.16, 4.56)	–	0.017	1.76 (0.98, 3.16)	–	0.059
Education (*High school or higher vs. less than high school*)	1.13 (0.44, 2.89)	–	0.798	1.10 (0.47, 2.53)	–	0.831
Income						
<$15,000 vs. $50,000 or more	22.67 (6.39, 80.43)	0.002	<0.0001	15.25 (5.30, 43.91)	0.003	<0.0001
$15,000 to less than $25,000 vs. $50,000 or more	22.99 (6.85, 77.12)	0.0004		11.81 (4.80, 29.06)	0.005	
$25,000 to less than $50,000 vs. $50,000 or more	8.09 (2.77, 23.63)	0.989		4.68 (2.31, 9.46)	0.458	
Employment status						
Employed for wages vs. unemployed	0.76 (0.26, 2.23)	0.436	0.252	0.81 (0.35, 1.88)	0.463	0.001
Self-employed vs. unemployed	0.24 (0.03, 1.75)	0.267		0.41 (0.12, 1.34)	0.043	
Out of work <1 year vs. unemployed	0.65 (0.14, 3.01)	0.784		6.36 (1.80, 22.54)	0.0001	
Retired vs. unemployed	0.45 (0.20, 0.99)	0.571		0.54 (0.26, 1.13)	0.016	
Marital status						
Widowed/divorced/separated vs. never married	1.25 (0.48, 3.29)	0.823	0.860	0.30 (0.14, 0.63)	0.010	0.006
Married/member of an unmarried couple vs. never married	1.33 (0.47, 3.72)	0.668		0.36 (0.16, 0.77)	0.134	
Number of children						
1 child vs. no children	2.07 (0.55, 7.88)	0.794	0.156	4.42 (1.77, 11.07)	0.110	0.001
2 or more children vs. no children	3.05 (0.94, 9.96)	0.209		4.54 (1.78, 11.63)	0.098	
Health insurance						
Medicaid vs. no coverage	0.40 (0.06, 2.67)	0.569	0.877	0.40 (0.10, 1.58)	0.989	0.041
Medicare vs. no coverage	0.40 (0.06, 2.54)	0.477		0.26 (0.08, 0.87)	0.096	
Other insurance vs. no coverage	0.50 (0.06, 4.56)	0.936		0.51 (0.13, 1.97)	0.489	
Private plan vs. no coverage	0.49 (0.08, 2.91)	0.873		0.20 (0.06, 0.64)	0.008	
Total number of chronic health conditions	1.39 (1.09, 1.77)	–	0.009	1.56 (1.28, 1.90)	–	<0.0001

## Discussion

In this study, the prevalence of food insecurity even before the COVID-19 pandemic was disproportionally experienced by cancer survivors residing in NYC compared to the ROS. The burden of food insecurity amongst this vulnerable population exceeds the national average (11%) by almost twofold and is higher than the overall state average (24.9%) [16]. While other studies have reported a prevalence of food insecurity among cancer survivors in the range of 4.0–26.2%, we are unaware of any studies that have described a similar geographic inequity with urban areas outpacing more suburban or rural areas [17–19]. Among cancer survivors, the most significant determinant of food insecurity during both periods was an annual household income of less than $25,000. Our findings of low-income and non-White race as independent factors that increase the likelihood of being food insecure align with previous studies [20].

While the goal of this study was to assess whether cancer survivors experienced higher rates of food insecurity because of the pandemic, we instead observed an interesting attenuation of the disparity related to the social construct of race and ethnicity accompanied by a lowering of the odds based on household income. This finding may have occurred due to the increased availability of food assistance programs, unemployment insurance, and stimulus payments across NYS [21–23], especially in NYC as the pandemic’s epicenter. Evaluations of NYS policies demonstrate that food policy reforms gained significant momentum during the COVID-19 pandemic. More than 300 policy provisions were implemented across the State during the state of emergency (March 2020–June 2021). About two-thirds of the policies focused on supporting food businesses and workers, while the other third targeted ensuring adequate food access (32%) for needy communities [24]. These additional state programs layered on top of federal programs like the Federal Pandemic Unemployment Compensation program may have buffered the most vulnerable populations. Evidence of social protection programs in other countries resulting in reductions in food insecurity because of the pandemic further demonstrates the capabilities of these programs to close critical gaps in food access created by social determinants such as poverty, race, and ethnicity [25–28].

During the pandemic, about 40% of cancer survivor survey respondents were of working age (18–64). While the overall percentage (2.84%) of cancer survivors in 2021 who reported they were out of work for less than a year was small, it represented a significant increase compared to the prevalence in 2019 (1.1%). Employed individuals diagnosed with cancer have a variety of postdiagnosis employment trajectories [29]. While some survivors may report their most significant limitations in the first year postdiagnosis, in at least one study, cancer survivors more than 11 years postdiagnosis reported nearly double the number of work absence days within the preceding year versus their colleagues with no cancer diagnosis [30]. Unfortunately, in 2019 and 2021, NYS did not include the optional module of years since the first cancer diagnosis. Thus, we were unable to factor that into the analysis. However, data from the 2020 BRFSS module demonstrates that about 43% of respondents in that year were diagnosed with cancer more than 10 years, 19% 6–10 years, 38% 5 years or less, and 18% were still actively undergoing treatment.

There are several study strengths and limitations to consider in the context of our findings. Strengths of the study include the use of survey data provided by the BRFSS, which provides population-based estimates of food insecurity that are generalizable to all NYS adults. Furthermore, the pre- and intra-pandemic data comparisons allow for further examination of secular trends in food insecurity. To our knowledge, this is the first study to examine this trend in association with cancer. Most recently, Crossa et al., using data from the 2017–2018 NYC Community Health Survey, explored the associations between food insufficiency, a more severe form of food insecurity, and the chronic health conditions of hypertension, diabetes, obesity, and depression [31]. Food insufficiency was associated with a higher odd for all chronic health conditions except obesity. Similarly, they found geographic inequities regarding the neighborhoods with the highest prevalence of food insufficiency being those areas with the “*greatest social and economic inequities associated with the legacy of structural racism.*” As recent findings have demonstrated that the Bronx has the highest rate of food insecurity (39%) in the state our findings of geographic inequities of food insecurity among cancer survivors across the state is not surprising.

Lastly, the sample size of the BRFSS permitted more nuanced examination of food insecurity patterns across social and demographic characteristics, as well as geography, yielding novel insights in subgroups that are at increased risk for experiencing food insecurity and its sequelae. Unfortunately, given the cross-sectional nature of the BRFSS, we are unable to examine the persistence of food insecurity within NYS households over time, or the trajectory of cancer patients’ illness. Furthermore, we are unable to ascertain the impacts of food insecurity on chronic disease prevention or management, including among cancer survivors, which may have clinical implications for individuals living with cancer. More longitudinal data is needed to fully elucidate the associations and pathways between food insecurity and health status, including poor diet quality, adverse mental health outcomes, or avoidance of medical treatment or medication adherence due to cost.

In light of the Biden Administration’s commitment coming out of the White House Conference on Hunger, Nutrition, and Health to Ending Hunger and Reducing Diet-Related Diseases and Disparities, continued research highlighting the deleterious effects of food insecurity on cancer risk, early detection, and cancer treatment response and outcomes, as well as on interventions to address food insecurity, are warranted [32]. Research is currently being conducted on the effectiveness of monthly unconditional guaranteed income for people with cancer and its impact on food insecurity and cancer outcomes [33]. Within NYC, interventions to address food insecurity through vouchers and linkages to food pantries have shown promise in improving cancer treatment completion [34]. NYC has also taken steps to institutionalize long-term food systems planning through the 10-year food policy plan, “Food Forward NYC,” mandated by local law LL 2020/040. The plan outlines five strategic areas for developing future food policy around food access and food security, food economy and good jobs, supply chains and regional infrastructure, environmental sustainability, and food system knowledge and governance [35].

Implementing ongoing, embedded food insecurity screening across all cancer care sites, at an evidence-based cadence to be determined, could serve as a cancer and food insecurity surveillance system and an indicator of the impact of food insecurity policies, which could be an essential policy intervention. This should be coupled with community-engaged interventions and advocacy for upstream policy approaches, including food voucher systems for low-income individuals, potentially coupled with Medicaid and Emergency Medicaid enrollment, to ensure progress toward addressing health inequities across the cancer continuum.

## Data Availability

The data underlying this article are available by request to the Behavioral Risk Factor Surveillance System Data New York State Department of Health Bureau of Chronic Disease Evaluation and Research. The data analysis code used for analysis is available by contacting the corresponding author.

## References

[1] Coleman-Jensen, A., Rabbitt, M. P., Gregory, C. A., & Singh, A. (2021). *Household food security in the United States in 2020, ERR-298*.

[2] Poverty Guidelines. (2023). Retrieved November 27, 2023, from https://aspe.hhs.gov/topics/poverty-economic-mobility/poverty-guidelines

[3] Rabbitt, M. P., Hales, L. J., Burke, M. P., & Coleman-Jensen, A. (2023). *Household food security in the United States in 2022*.

[4] Simmons, L. A., Modesitt, S. C., Brody, A. C., & Leggin, A. B. (2006). Food insecurity among cancer patients in Kentucky: A pilot study. *Journal of Oncology Practice,**2*(6), 274–279. 10.1200/jop.2006.2.6.274 20859354 10.1200/jop.2006.2.6.274 PMC2793655

[5] Center for Disease Prevention and Control. (2022). *BRFSS prevalence & trends data. Behavioral Risk Factor Surveillance System*. Center for Disease Prevention and Control.

[6] Expanded Behavioral Risk Factor Surveillance System (Expanded BRFSS). Retrieved November 27, 2023, from https://www.health.ny.gov/statistics/brfss/expanded/

[7] New York State Department of Health. Retrieved from https://www.health.ny.gov/statistics/brfss/expanded/

[8] New York State Department of Health. Retrieved from https://www.health.ny.gov/statistics/brfss/reports/docs/survivorship_compendium_2018.pdf

[9] Complex Sampling Weights and Preparing 2021 BRFSS Module Data for Analysis. (2022). Retrieved November 27, 2023, from https://www.cdc.gov/brfss/annual_data/2021/pdf/Complex-Sampling-Weights-and-Preparing-Module-Data-for-Analysis-2021-508.pdf

[10] Complex Sampling Weights and Preparing 2019 BRFSS Module Data for Analysis. (2020). Retrieved November 27, 2023, from https://www.cdc.gov/brfss/annual_data/2019/pdf/complex-smple-weights-prep-module-data-analysis-2019-508.pdf

[11] Cai, J., & Bidulescu, A. (2023). The association between chronic conditions, COVID-19 infection, and food insecurity among the older US adults: Findings from the 2020–2021 National Health Interview Survey. *BMC Public Health,**23*, 179. 10.1186/s12889-023-15061-8 36703149 10.1186/s12889-023-15061-8 PMC9880360

[12] Camacho-Rivera, M., Albury, J., Chen, K., Ye, Z., & Islam, J. Y. (2022). Burden of food insecurity and mental health symptoms among adults with cardiometabolic conditions during the COVID-19 pandemic. *International Journal of Environmental Research and Public Health,**19*, 16. 10.3390/ijerph191610077 10.3390/ijerph191610077 PMC9408010 36011710

[13] Ni, K., Wan, Y., & Zheng, Y. (2023). Association between adult food insecurity and self-reported asthma in the United States: NHANES 2003–2018. *Journal of Asthma,**60*(11), 2074–2082. 10.1080/02770903.2023.2214921 10.1080/02770903.2023.2214921 37255268

[14] Seligman, H. K., Bindman, A. B., Vittinghoff, E., Kanaya, A. M., & Kushel, M. B. (2007). Food insecurity is associated with diabetes mellitus: Results from the National Health Examination and Nutrition Examination Survey (NHANES) 1999–2002. *Journal of General Internal Medicine,**22*(7), 1018–23. 10.1007/s11606-007-0192-6 17436030 10.1007/s11606-007-0192-6 PMC2583797

[15] Seligman, Hilary K., Laraia, Barbara A., & Kushel, Margot B. (2011). ERRATUM: Food insecurity is associated with chronic disease among low-income NHANES participants 1, 2. *Journal of Nutrition,**140*(2), 304–310. 10.3945/jn.109.112573 10.3945/jn.109.112573 PMC2806885 20032485

[16] Self-Reported Food Insecurity Among New York State Adults by County, BRFSS 2021: 2023.

[17] Beavis, A. L., Sanneh, A., Stone, R. L., Vitale, M., Levinson, K., Rositch, A. F., Fader, A. N., Topel, K., Abing, A., & Wethington, S. L. (2020). Basic social resource needs screening in the gynecologic oncology clinic: A quality improvement initiative. *American Journal of Obstetrics and Gynecology,**223*(5), 735.e1-735.e14. 10.1016/j.ajog.2020.05.028 32433998 10.1016/j.ajog.2020.05.028 PMC8340269

[18] Byker Shanks, C., Andress, L., Hardison-Moody, A., Jilcott Pitts, S., Patton-Lopez, M., Prewitt, T. E., Dupuis, V., Wong, K., Kirk-Epstein, M., Engelhard, E., Hake, M., Osborne, I., Hoff, C., & Haynes-Maslow, L. (2022). Food insecurity in the rural United States: An examination of struggles and coping mechanisms to feed a family among households with a low-income. *Nutrients,**14*(24), 5250. 10.3390/nu14245250 36558409 10.3390/nu14245250 PMC9785039

[19] Poghosyan, H., & Scarpino, S. V. (2019). Food insecure cancer survivors continue to smoke after their diagnosis despite not having enough to eat: Implications for policy and clinical interventions. *Cancer Causes and Control,**30*(3), 241–248. 10.1007/s10552-019-01137-7 30729359 10.1007/s10552-019-01137-7

[20] Robien, K., Clausen, M., Sullo, E., Ford, Y. R., Griffith, K. A., Le, D., Wickersham, K. E., & Wallington, S. F. (2023). Prevalence of food insecurity among cancer survivors in the United States: A scoping review. *Journal of the Academy of Nutrition and Dietetics,**123*(2), 330–346. 10.1016/j.jand.2022.07.004 35840079 10.1016/j.jand.2022.07.004 PMC12323884

[21] Kumar, S. L., Calvo-Friedman, A., Freeman, A. L., Fazio, D., Johnson, A. K., Seiferth, F., Clapp, J., Davis, N. J., & Schretzman, M. (2023). An unconditional cash transfer program for low-income New Yorkers affected by COVID-19. *Journal of Urban Health,**100*(1), 16–28. 36224486 10.1007/s11524-022-00693-9 PMC9555690

[22] Morita, S. X., & Kato, H. (2023). Racial disparity and trend of food scarcity amid COVID-19 pandemic in the United States. *Cureus*. 10.7759/cureus.33232 36733557 10.7759/cureus.33232 PMC9889840

[23] Winkler, L., Goodell, T., Nizamuddin, S., Blumenthal, S., & Atalan-Helicke, N. (2023). The COVID-19 pandemic and food assistance organizations’ responses in New York’s Capital District. *Agriculture and Human Values,**40*(3), 1–15. 10.1007/s10460-022-10400-8 10.1007/s10460-022-10400-8 PMC9734295 36530206

[24] Ilieva, R. T., Fraser, K. T., & Cohen, N. (2023). From multiple streams to a torrent: A case study of food policymaking and innovations in New York during the COVID-19 emergency. *Cities,**136*, 104222. 10.1016/j.cities.2023.104222 36879669 10.1016/j.cities.2023.104222 PMC9977326

[25] Parliament of Australia. (2022). *Australian Government COVID-19 Disaster Payments: A quick guide*.

[26] Béland, D., Dinan, S., Rocco, P., & Waddan, A. (2021). Social policy responses to COVID-19 in Canada and the United States: Explaining policy variations between two liberal welfare state regimes. *Social Policy and Administration,**55*, 280–294.

[27] Lustig, N., & Trasberg, M. (2021). How Brazil and Mexico diverged on social protection in the pandemic. *Current History,**120*(823), 57–63.

[28] Palma, J., & Araos, C. (2021). Household coping strategies during the COVID-19 pandemic in Chile. *Frontiers in Sociology.,**6*, 728095. 10.3389/fsoc.2021.728095 34532353 10.3389/fsoc.2021.728095 PMC8439567

[29] Nekhlyudov, L., Walker, R., Ziebell, R., Rabin, B., Nutt, S., & Chubak, J. (2016). Cancer survivors’ experiences with insurance, finances, and employment: Results from a multisite study. *Journal of Cancer Survivorship,**10*(6), 1104–1111. 10.1007/s11764-016-0554-3 27277896 10.1007/s11764-016-0554-3

[30] Yabroff, K. R., Lawrence, W. F., Clauser, S., Davis, W. W., & Brown, M. L. (2004). Burden of illness in cancer survivors: Findings from a population-based national sample. *Journal of the National Cancer Institute,**96*(17), 1322–30. 10.1093/jnci/djh255 15339970 10.1093/jnci/djh255

[31] Crossa, A., Leon, S., Prasad, D., & Baquero, M. C. (2024). Associations between food insufficiency and health conditions among New York city adults, 2017–2018. *Journal of Community Health*. 10.1007/s10900-023-01296-4 38407756 10.1007/s10900-023-01296-4

[32] Maino Vieytes, C. A., Zhu, R., Gany, F., Burton-Obanla, A., & Arthur, A. E. (2022). Empirical dietary patterns associated with food insecurity in U.S. cancer survivors: NHANES 1999–2018. *International Journal of Environmental Research and Public Health,**19*(21), 14062. 10.3390/ijerph192114062 36360938 10.3390/ijerph192114062 PMC9656362

[33] Doherty, M., Heintz, J., Leader, A., Wittenberg, D., Ben-Shalom, Y., Jacoby, J., Castro, A., & West, S. (2023). Guaranteed Income and Financial Treatment (G.I.F.T.): A 12-month, randomized controlled trial to compare the effectiveness of monthly unconditional cash transfers to treatment as usual in reducing financial toxicity in people with cancer who have low incomes. *Frontiers in Psychology,**14*, 1179320. 10.3389/fpsyg.2023.1179320 37275728 10.3389/fpsyg.2023.1179320 PMC10234289

[34] Gany, F., Melnic, I., Wu, M., Li, Y., Finik, J., Ramirez, J., Blinder, V., Kemeny, M., Guevara, E., Hwang, C., & Leng, J. (2022). Food to overcome outcomes disparities: A randomized controlled trial of food insecurity interventions to improve cancer outcomes. *Journal of Clinical Oncology,**40*(31), 3603–3612. 10.1200/JCO.21.02400 35709430 10.1200/JCO.21.02400 PMC9622577

[35] (2022). *Food forward NYC: A 10 year food plan*. Retrieved April 15, 2024, from https://www.nyc.gov/site/foodpolicy/reports-and-data/food-forward.page

